# Borderline breast core needle histology: predictive values for malignancy in lesions of uncertain malignant potential (B3)

**DOI:** 10.1038/sj.bjc.6603714

**Published:** 2007-04-17

**Authors:** N Houssami, S Ciatto, M Bilous, V Vezzosi, S Bianchi

**Affiliations:** 1 Department of Diagnostic Imaging, Centro per lo Studio e la Prevenzione Oncologica (CSPO), Viale A. Volta 171, 50131 Florence, Italy; 2 Screening and Test Evaluation Program (STEP), School of Public Health, University of Sydney, Sydney, Australia; 3 Department of Tissue Pathology, Institute of Clinical Pathology and Medical Research (ICPMR), Westmead Hospital, NSW, Australia; 4 Department of Human Pathology and Oncology, Universitá delgi Studi di Firenze, Viale Morgagni, 85, 50134 Florence, Italy

**Keywords:** breast neoplasm, core needle biopsy, borderline histology, atypical hyperplasia, predictive value

## Abstract

Breast core needle biopsy (CNB) is an accurate test but may result in borderline histology (lesions of uncertain malignant potential or B3). This is an evaluation of the largest series (to date) of B3 histology, which focusses on estimating positive predictive values (PPV) for malignancy. We identified all B3 CNBs over a 10-year period in a single institution (*N*=372) from a series of 4035 consecutive needle biopsies. We describe the imaging findings, and report excision histology outcomes (*N*=279) and category-specific PPV for B3 lesions using two approaches including estimates based on subjects who had either excision or follow-up (*N*=328). B3 represented 9.2% of all CNB results. Excision histology was benign in 181 (64.9%) and malignant in 98 (35.1%) subjects (61 ductal carcinoma *in situ*, 37 invasive carcinoma). Positive predictive value for malignancy (based on excision histology) was 35.1% (95% CI: 29.5–40.7) and PPV (based on excision or review) was 29.9% (95% CI: 24.9–34.8). Lesion-specific PPV (estimates in parentheses for excision or follow-up) was atypical ductal hyperplasia 44.7% (40.6%); lobular intraepithelial neoplasia 60.9% (58.3%); papillary lesion 22.7% (15.9%); radial scar 16.7% (12.3%); phyllodes tumour 12.5% (12.5%); and B3 not specified 20.0%. Approximately one-third of CNB results classified as B3 are malignant on excision, and the likelihood of malignancy varies substantially between specific lesion groups. Whereas cases may be selectively managed without surgery, the majority warrant excision biopsy based on our estimates. Research is needed to improve differentiation between malignant and benign diseases in B3 lesions using diagnostic or predictive methods.

Percutaneous core needle biopsy (CNB) is an accurate test for sampling breast lesions (Parker *et al*, 1994; Burbank, 1997; Liberman, 2000; Ciatto *et al*, 2006) and is increasingly replacing fine needle aspiration cytology (FNAC) in breast diagnosis. Core needle biopsy has advantages over FNAC in that it provides histological information, which improves sensitivity and may assist pre-operative treatment planning (Houssami *et al*, 2006), and is rarely associated with inadequacy (insufficiency) (Ciatto *et al*, 2006). It also has a complementary role to FNAC where FNAC is used as an initial test and where the cytology is atypical (Bulgaresi *et al*, 2006) – subsequent use of CNB in this context can establish a definitive diagnosis. The major limitation that has emerged with CNB is the borderline histology spectrum, a heterogeneous group of lesions also described as lesions of uncertain malignant potential (B3 core histology) in international standards (NHS Non-operative Diagnosis Subgroup of the National Coordination Group for Breast Screening Pathology, 2001; Perry *et al*, 2006).

Despite the current perspective that CNB has better diagnostic discrimination between benign and malignant breast disease than FNAC, borderline (B3) core needle histology occurs in a similar (Lee *et al*, 2003) or higher proportion of cases as does atypical or borderline cytology (when considered within the same institutional context) (Ciatto *et al*, 1993; Lee *et al*, 2003; Ciatto *et al*, 2006). Borderline (B3) core needle histology, however, has more significant implications in that most cases progress to surgical intervention, and debate continues on whether selected cases may be spared excision (Jacobs *et al*, 2002). While most cases of B3 CNB will in fact have benign disease, approximately one-quarter (Lee *et al*, 2003) or more will have cancer on excision; however, estimates of the proportion with malignancy on excision biopsy are based on small series (Jacobs *et al*, 2002).

We present a retrospective evaluation of the largest series, to date, of borderline (B3) core needle histology, and report on excision histology outcomes. We provide B3 (lesions of uncertain malignant potential) subcategory-specific positive predictive values (PPV) for malignancy (*in situ* and invasive), and discuss implications for practice and future research.

## MATERIALS AND METHODS

From all subjects who attended the study centre between January 1996 and March 2005, and had a core biopsy of any type (4035 biopsies), we identified eligible subjects as all those with B3 core histology reports (*N*=372). We have included all eligible cases in our descriptive evaluation, and for histological outcomes we report on subjects who had excision histology (*N*=279). To avoid verification bias in estimating category-specific PPV (Houssami and Irwig, 1998), we have retained in the PPV analysis all eligible subjects who had either excision histology or at least one episode of follow-up (*N*=328). Imaging lesions were considered stable on follow-up if unchanged on imaging (and clinical) review at 6–12 months (for masses) or a minimum of 12 months (for microcalcification) (Ciatto *et al*, 2006).

Detailed methodology of all consecutive core biopsies, from which this series of borderline core needle histology originated, has been published in our companion paper (Ciatto *et al*, 2006). In brief, CNB was performed over the timeframe of the study by one of 13 radiologists who also performed the clinical examination. Core needle biopsy was used for imaging lesions that were atypical or associated with some degree of suspicion. At the study centre, BI-RADS is not routinely used for reporting imaging, an alternate classification is used with published estimates of the likelihood of malignancy (Houssami and Irwig, 1998). Most lesions in this series were therefore imaging categories 3–5 (3 – indeterminate, 4 – suspicious and 5 – malignant) (Houssami and Irwig, 1998). Core needle biopsy in the study centre was generally performed using conventional automated (14 G) core needle for sampling masses, whereas microcalcifications were preferentially sampled using vacuum-assisted biopsy (11G) and less often using automated (14 G) core (Ciatto *et al*, 2006). Stereotactic sampling of microcalcification was verified with specimen X-ray. Ultrasound guidance was preferentially used for all lesions clearly seen on ultrasound and also for masses (including where palpable), and the use of stereotaxis was largely for microcalcification and impalpable mammographic lesions.

One of two breast pathologists reported all core needle histology (without knowledge of outcomes), with one pathologist (SB) interpreting the vast majority (>90%). Results were reported in line with UK and European guidelines (NHS Non-operative Diagnosis Subgroup of the National Coordination Group for Breast Screening Pathology, 2001; Perry *et al*, 2006). Categories are B1=normal tissue/unsatisfactory, B2=benign, B3=lesions of uncertain malignant potential, B4=suspicious of malignancy, B5 (malignant–sub-classified as ductal carcinoma *in situ* (DCIS) or invasive cancer) (NHS Non-operative Diagnosis Subgroup of the National Coordination Group for Breast Screening Pathology, 2001; Perry *et al*, 2006). The borderline histology or B3 category includes atypical ductal hyperplasia (ADH), lobular intraepithelial neoplasia (LIN) (regrouping of the former atypical lobular hyperplasia (ALH) and lobular carcinoma *in situ* (LCIS)), papillary lesions, radial scar/complex sclerosing lesion, mucocoele-like lesion and phyllodes tumour.

We describe the distribution of outcomes, the type of imaging lesion and whether clinically palpable, in all subjects. We also provide a descriptive analysis of imaging findings for all subjects according to B3 groups and in relation to outcomes to provide clinical context. Excision histology findings are reported for benign and malignant outcomes, and PPVs are calculated for all B3 core histology and for each subcategory. We have estimated PPVs using two approaches, one based on all cases verified with excision histology and also cases shown to have remained stable on review (this minimises verification bias in estimating measures of accuracy) (Houssami and Irwig, 1998). We have also calculated them using the more conventional approach of including only those verified with excision histology.

## RESULTS

B3 core needle histology comprised 9.2% 372 from 4035 of all CNB results. To assist in judging generalisability of our series, we have presented in Table 1 the distribution of all B3 cases according to type of lesion depicted on mammography, excision histology or other outcomes and palpability. Microcalcification (46%) and masses (44.6%) were about equally distributed, and distortions represented 9.4% of lesions assessed with CNB. Imaging lesions were clinically palpable in 27.4% of cases, and 72.6% were considered impalpable (Table 1), reflecting the high frequency of isolated microcalcification evaluated with CNB (all of which were impalpable). Data in Table 1 included six cases of suspicious (B3–B4) results, which on further review were considered to represent B4, and were therefore excluded from our analysis of PPV – all six cases were found to represent malignancy (one DCIS, five invasive cancers). A detailed distribution of all B3 core needle histology cases according to the type of lesion depicted on imaging and the level of suspicion on imaging, in relation to outcomes, is included as Appendix A.

Table 2 summarises all excision histology outcomes for B3 core needle histology, overall and for each subcategory of B3, and presents category-specific PPV. Overall, PPV for malignancy (based on excision histology) was 35.1% (95% CI: 29.5–40.7) and PPV (based on excision or review) was 29.9% (95% CI: 24.9–34.8). There were five cases of B3 not otherwise specified based on the original pathology report (Table 2). These were subsequently reviewed and were classified as B3 phyllodes, B3 ADH/columnar cell lesion, B3 fibromatosis or phyllodes, B3 granular cell tumour and B2 fibroadenolipoma.

## DISCUSSION

We have reported B3 (borderline lesions or lesions of uncertain malignant potential) category-specific PPV for malignancy (DCIS and invasive); B3 core needle histology comprised about 9% of all consecutive CNB performed in the study centre. We are not aware of any published work that estimates PPV for subcategories of B3 lesions in one comprehensive series. Overall, about one-third of borderline lesions on CNB in this study proved to be malignant (PPV 29.9 or 35.1%); however, the PPV for each subcategory within B3 varied substantially (Table 2). In an overview of this topic, Jacobs *et al* (2002) reported that knowledge and consensus on the management of these lesions are hindered by the very small number of subjects in most studies, and also by selection bias for subjects managed with excision biopsy (Jacobs *et al*, 2002). Whereas acknowledging that a few studies have included more cases per specific B3 lesion (Cawson *et al*, 2003; Shah *et al*, 2006), we believe that our study addresses the first issue in providing the largest consecutive study of B3 cases. As for the latter issue, and while selection to surgery was not entirely avoided, the vast majority of subjects had excision in our series. We have also included outcomes for some of the subjects that did not have surgery, and overall, we have reported outcomes in approximately 88% of subjects.

We have explained the reasons we report two estimates of PPV – methodologies that include all subjects with known outcomes (and not only those verified on the basis of excision) have been shown to reduce verification bias in calculating measures of accuracy (Houssami and Irwig, 1998), whereas subjects with outcomes verified on the basis of excision histology are traditionally included to avoid misclassification. We have provided estimates of PPV using both methods, and acknowledge the limitation of length of follow-up in the minority group that had follow-up only in our series. It should be noted that the proportion of CNB reported to be B3 and the distribution of lesion categories within the borderline CNB spectrum will vary between institutions, as this is dependent on the criteria used for reporting as well as the number of pathologists involved in reporting and their threshold for classifying a lesion as borderline on CNB. We also point out that overall and lesion-specific predictive values may vary in different settings according to the type of imaging abnormalities assessed with CNB and the sampling methods used; we have described the study centre's practice in this paper and in our related paper (Ciatto *et al*, 2006), and the data in Appendix A provide detailed imaging context for the series.

Lee *et al* (2003) provide data on histological outcomes for the B3 category in 96 excised lesions, and report an overall PPV of 26% for B3 CNB histology that is slightly lower than our overall estimates. These authors make the point that excision biopsy findings for B3 are heterogeneous, that the clinical and radiological features are important in deciding further management (and that this should be carried out in multidisciplinary meetings). We agree with these views, and our work highlights the heterogeneity of the borderline or B3 CNB category, in addition to evidence on lesion-related variability in likelihood of malignancy as an outcome in B3 cases. Clearly, lesions categorised on CNB as ADH (more appropriately termed atypical intraductal epithelial proliferation on CNB histology), or as LIN, are associated with a significantly higher probability of cancer as an outcome relative to the ‘other’ borderline lesions. It may be worthwhile reconsidering current categorisation criteria (NHS Non-operative Diagnosis Subgroup of the National Coordination Group for Breast Screening Pathology, 2001; Perry *et al*, 2006) and potentially modifying the B3 category into two risk-based subgroups (B3 with lower probability of cancer, B3 with higher probability of cancer). This would be expected to improve specificity and may assist management decisions in the context of team discussion.

The most frequent lesion in our B3 series is ADH, and its frequency within the B3 category is similar to that reported by Lee *et al* (2003). The PPV for ADH (44.7 or 40.6%) is also similar to other studies (Jackman *et al*, 1999; Harvey *et al*, 2002; Lee *et al*, 2003), although we have reported estimates based on a larger number of cases. This estimate further emphasises that the standard of care for lesions yielding ADH on CNB should be excision biopsy.

Our PPV estimates show the highest lesion-specific risk of malignancy in association with LIN (ALH and LCIS) with a higher likelihood of malignancy for this category than reported in other series (Lee *et al*, 2003; Elsheikh and Silverman, 2005). The evidence on, and the management of, lobular neoplasia continues to be contradictory and controversial (Lakhani *et al*, 2006) and a detailed discussion of this lesion category is beyond the scope of this paper. The largest published series that examined pure lobular neoplasia (based on 33 subjects) emphasised that all CNB diagnoses of ALH and LCIS warrant surgical excision because of a high risk of malignancy on excision histology (Elsheikh and Silverman, 2005). We also point out that most other series of lobular neoplasia on CNB have been based on very few subjects with many subjects not progressing to excision biopsy (Jacobs *et al*, 2002). The vast majority of cases of lobular neoplasia on CNB in our series were managed with surgery and most were shown to have DCIS on excision histology – since many of the CNBs were performed for imaging lesions, some of our cases are likely to have represented under-sampling of the target lesion. It is particularly important that CNB reports of LIN be correlated with the clinical and imaging findings, and that management decisions are decided on the basis of team discussion (Lee *et al*, 2003; Lakhani *et al*, 2006).

Unlike our PPV for papillary lesions (22.7 or 15.9%), one study of screen-detected papillary lesions reported benign excision histology outcomes in all 15 CNB diagnoses of ‘papillary lesion’ classified as B3 (Carder *et al*, 2005). Liberman *et al* (2006) evaluated 35 cases with a diagnosis of (benign) papilloma on CNB (and concordant with imaging), and reported a cancer yield of 14% (based on either surgery or follow-up), which is similar to our PPV for papillary lesion on CNB (based on either surgery or follow-up). A review by Jacobs *et al* (2002) reported malignancy as an outcome following a CNB diagnosis of papillary lesion (without atypia) ranging between 0 and 25%. Shah *et al* (2006) reported improved accuracy for categorising benign papillary lesions on CNB by using immunohistochemistry. Variability in reported estimates for this lesion (and other lesions in the borderline category) may be due to a combination of factors as we have already discussed, including the proportion of subjects with verified outcomes, selection of imaging lesions assessed with core biopsy, study sample size and pathology reporting methods.

The lesions with the lowest PPV for malignancy in our study are phyllodes tumour and radial scar. The recommended management of both these lesions is surgical excision to differentiate phyllodes tumour from fibroadenoma in the former and to exclude associated malignancy in the latter. Research efforts might particularly target lesions with a lower PPV for malignancy in exploring selective non-surgical management. Because of the timeframe of our study, columnar cell lesions were not included as a separate category; however, the potential importance of columnar cell lesions and columnar cell atypia in predicting malignancy is another area for future research.

We have described the histology in lesions with benign outcomes on excision – these data show moderate concordance between the specific benign histology reported on CNB and excision biopsy, and we have included the number of cases upgraded to lesions with increased future risk of breast cancer. It would be reasonable to argue that such distinctions within the benign pathology spectrum are of limited clinical relevance, but we have provided the information to be comprehensive as this may be relevant to clinicians providing care and surveillance advice to women with benign breast disease.

Our work emphasises the heterogeneity of lesions of uncertain malignant potential (B3) as far as risk of malignancy. Research is needed to help modify our current approach of surgical management for almost all borderline core needle histology, thus minimising unnecessary surgery in the majority of women with B3 on CNB and have benign disease. One example is optimising criteria for reporting lesions as B3 on CNB – pathologists might work on characterisation of morphological features that potentially allow accurate shifting of some B3 lesions into either the benign or the malignant category. Another area in need of formal evaluation is the selective use of large (vacuum-assisted) core biopsy devices in lesions deemed to be low risk (low PPV) on CNB to either re-sample or remove the imaging lesion, as an alternate to surgical excision. This should only be performed in the context of radiology–pathology correlation and team discussion to ensure appropriate selection of low risk lesions for this form of management.

The most relevant research, however, is likely to be in potentially identifying factors or methods that accurately predict outcomes on excision histology for the spectrum of B3 lesions. This may be achieved through two approaches, the first being the evaluation of molecular or genetic markers as indicators of malignancy. Although genetic profiling has indicated that current histological classification of breast cancer does not predict either lymph node metastases or response to therapy, it remains possible that the combination of genetic profile and core needle histology could provide a new diagnostic modality with the greatest discriminatory ability for outcomes. The second approach is the development of predictive systems based on clinical–radiological–CNB correlates with excision histology. We are currently exploring the latter of these approaches.

## Figures and Tables

**Table 1:** Distribution of B3 (lesions of uncertain malignant potential) core needle histology (*N*=372): type of lesion depicted on mammography, outcomes and palpability

**Lesion type on mammography**	**Excision histology outcome (*N*=285)^a^**				**Lesions not excised**		**Total (number palpable)^c^**	
	**Benign**	**Atypical hyperplasias^b^**	**DCIS**	**Invasive cancer**	**Stable on review**	**Pending review**		
Mass (or density)	53	31	15	20	31	16	166	(98)
Distortion	21	3	1	3	5	2	35	(4)
Microcalcification	39	34	46	19	13	20	171	(0)
Total number	113	68	62	42	49	38	372	(102)

DCIS=ductal carcinoma *in situ*.

a This included six cases of suspicious CNB results (retained only for descriptive analysis).

b ‘Atypical hyperplasias’ included atypical ductal hyperplasia and lobular intraepithelial neoplasia (lesions associated with increased future risk of breast cancer).

c Number palpable=number of lesions from total (and for each type of mammographic lesion) considered to be evident on clinical examination.

**Table 2:** Lesions of uncertain malignant potential (B3) on CNB: excision histology outcomes for different B3 lesions and associated PPV for breast malignancy

**Subcategory of B3 core needle histology (number of cases with excision outcomes)**	**Benign on excision histology**				**Invasive cancer**	**DCIS**	**PPV % (excision or review)^b^**		**PPV % (excision histology)^c^**	
	**Number (% of all excised lesions)**		**Number with concordant histology (number upstaged to a high risk lesion^a^)**							
ADH^d^ (141)	78	(55.3)	42	(NA)	21	42	40.6	(63/155)	44.7	(63/141)
LIN (23)	9	(39.1)	3	(NA)	2	12	58.3	(14/24)	60.9	(14/23)
Papillary lesion (44)	34	(77.3)	7	(6)	7	3	15.9	(10/63)	22.7	(10/44)
Radial scar (42)	35	(83.3)	15	(5)	3	4	12.3	(7/57)	16.7	(7/42)
Phyllodes tumour (24)	21	(87.5)	16	(0)	3	0	12.5	(3/24)	12.5	(3/24)
B3 (not otherwise specified) (5)	4	(80.0)	NA	(0)	1	0	20.0	(1/5)	20.0	(1/5)
All B3 categories (279)	181	(64.9)	83	(11)	37	61	29.9	(98/328)	35.1	(98/279)

ADH=atypical ductal hyperplasia; DCIS=ductal carcinoma *in situ*; LIN=lobular intraepithelial neoplasia; NA=not applicable; PPV=positive predictive values.

a Where excision histology specifies ADH or lobular neoplasia (lesions with known long-term risk of breast cancer) as the dominant lesion.

b PPV based on all cases verified with excision histology and cases shown to have remained stable on follow-up (*N*=328).

c PPV based on all cases verified with excision histology (*N*=279).

d ADH on CNB is more appropriately described as atypical intraductal epithelial proliferation.

**Table A1:** Distribution of B3 (lesions of uncertain malignant potential) core needle histology cases according to the type of lesion depicted on imaging^a^ and the level of suspicion on imaging^b^ in relation to outcomes in all subjects (*N*=372)

**Subcategory of B3 core needle histology (number of cases)**	**Lesions with excision histology outcome (*n*=285)^c^**		**Lesions not excised (*n*=87) (stable on review (49) or pending review (38))**
	**Benign including atypical hyperplasias^d^ (*n*=181)**	**Malignant (DCIS and invasive cancer) (*n*=104)**	
ADH	M=20 (IM3=11, IM4=9)	M=14 (IM3=5, IM4=8, IM5=1)	M=5 (IM3=5)
(*N*=172)	D=3 (IM3=1, IM4=2)	D=1 (IM4=1)	D=3 (IM3=3)
	C=55 (IM3=22, IM4=31, IM5=2)	C=48 (IM3=19, IM4=26, IM5=3)	C=23 (IM3=9, IM4=13, IM5=1)
LIN	M=2 (IM3=1, IM4=1)	M=4 (IM4=2, IM5=2)	M=2 (IM2=2)
(*N*=29)	D=3 (IM3=1, IM4=2)	D=0	D=0
	C=4 (IM3=2, IM4=2)	C=10 (IM3=6, IM4=4)	C=4 (IM3=1, IM4=3)
Papillary lesion	M=26 (IM3=20, IM4=6)	M=7 (IM3=6, IM4=1)	M=22 (IM3=18, IM4=4)
(*N*=70)	D=0	D=0	C=4 (IM3=3, IM4=1)
	C=8 (IM3=6, IM4=2)	C=3 (IM3=1, IM4=2)	
Radial scar	M=12 (IM3=4, IM4=7, IM5=1)	M=3 (IM3=1, IM4=2)	M=15 (IM3=6, IM4=9)
(*N*=63)	D=19 (IM3=12, IM4=7)	D=2 (IM4=2)	D=4 (IM3=3, IM4=1)
	C=4 (IM3=3, IM4=1)	C=2 (IM3=1, IM4=1)	C=2 (IM4=2)
Phyllodes tumour	M=21 (IM3=20, IM4=1)	M=3 (IM3=3)	M=1 (IM3=1)
(*N*=25)	D=0	D=0	D=0
	C=0	C=0	C=0
B3 (not otherwise specified)	M=2 (IM3=2)	M =1 (IM4=1)	M=1 (IM3=1)
(*N*=7)	D=0	D=0	D=0
	C=2 (IM4=2)	C=0	C=1 (IM4=1)
Categorised as suspicious	M=0	M=5 (IM3=4, IM4=1)	M=0
(B4)^c^ on review	D=0	D=1 (IM4=1)	D=0
(*N*=6)	C=0	C=0	C=0
All categories	M=83 (IM3=58, IM4=24, IM5=1)	M=37 (IM3=21, IM4=13, IM5=3)	M=46 (IM3=33, IM4=13)
(*N*=372)	D=25 (IM3=14, IM4=11)	D=4 (IM3=4)	D=7 (IM3=6, IM4=1)
	C=73 (IM3=33, IM4=38, IM5=2)	C=63 (IM3=27, IM4=33, IM5=3)	C=34 (IM3=13, IM4=20, IM5=1)

ADH=atypical ductal hyperplasia; LIN=lobular intraepithelial neoplasia.

a Type of lesion depicted on imaging: M=mass (or density), D=distortion, C=microcalcacification.

b Level of suspicion on imaging (IM) using categories 1–5 (Houssami and Irwig, 1998) based on mammography classification, or on ultrasound classification where mammography was not carried out or was negative.

c Six cases were classified as suspicious CNB results on further review (retained only for descriptive analysis).

d ‘Atypical hyperplasias’ on excision histology included ADH and LIN (lesions associated with increased future risk of breast cancer).

## Appendix A

Table A1

## References

[bib1] Bulgaresi P, Cariaggi P, Ciatto S, Houssami N (2006) Positive predictive value of breast fine needle aspiration cytology (FNAC) in combination with clinical and imaging findings: a series of 2,334 subjects with abnormal cytology. Breast Cancer Res Treat 97: 319–321, doi: 10.1007/s10549-005-9126-3 16570117 10.1007/s10549-005-9126-3

[bib2] Burbank F (1997) Stereotactic breast biopsy of atypical ductal hyperplasia and ductal carcinoma *in situ* lesions: improved accuracy with a directional, vacuum-assisted biopsy instrument. Radiology 202: 843–847 9051043 10.1148/radiology.202.3.9051043

[bib3] Carder PJ, Garvican J, Haigh I, Liston JC (2005) Needle core biopsy can reliably distinguish between benign and malignant papillary lesions of the breast. Histopathology 46: 320–327, doi:10.1111/j.1365-2559.2005.02082.x 15720418 10.1111/j.1365-2559.2005.02082.x

[bib4] Cawson JN, Malara F, Kavanagh A, Hill P, Balasubramanium G, Henderson M (2003) Fourteen-gauge needle core biopsy of mammographically evident radial scars. Is excision necessary? Cancer 97: 345–351 12518358 10.1002/cncr.11070

[bib5] Ciatto S, Cariaggi P, Bulgaresi P, Confortini M, Bonardi R (1993) Fine needle aspiration cytology of the breast: review of 9533 consecutive cases. Breast 2: 87–90, doi: 10.1016/0960-9776(93)90163-A

[bib6] Ciatto S, Houssami N, Ambrogetti D, Bianchi S, Bonardi R, Brancato B, Catarzi S, Risso G (2006) Accuracy and underestimation of malignancy of breast core needle biopsy: the Florence experience of over 4,000 consecutive biopsies. Breast Cancer Res Treat 101: 291–297, doi: 10.1007/s10549-006-9289-6 16823506 10.1007/s10549-006-9289-6

[bib7] Elsheikh TM, Silverman JF (2005) Follow-up surgical excision is indicated when breast core needle biopsies show atypical lobular hyperplasia or lobular carcinoma *in situ*: a correlative study of 33 patients with review of the literature. Am J Surg Pathol 29: 534–543 15767810 10.1097/01.pas.0000152566.78066.d1

[bib8] Harvey JM, Sterrett GF, Frost FA (2002) A typical ductal hyperplasia and atypia of uncertain significance in core biopsies from mammographically detected lesions: correlation with excision diagnosis. Pathology 34: 410–416, doi: 10.1080/0031302021000009315 12408338 10.1080/0031302021000009315

[bib9] Houssami N, Cuzick J, Dixon JM (2006) The prevention, detection and management of breast cancer. Med J Aust 184: 230–234 16515434 10.5694/j.1326-5377.2006.tb00208.x

[bib10] Houssami N, Irwig L (1998) Likelihood ratios for clinical examination, mammography, ultrasound and fine needle biopsy in women with breast problems. Breast 7: 85–89, doi: 10.1016/S0960-9776(98)90062-5

[bib11] Jackman RJ, Nowels KW, Rodriguez-Soto J, Marzoni Jr FA, Finkelstein SI, Shepard MJ (1999) Stereotactic automated large-core needle biopsy of non-palpable breast lesions: false-negative and histologic underestimation rates after long-term follow-up. Radiology 210: 799–805 10207484 10.1148/radiology.210.3.r99mr19799

[bib12] Jacobs TW, Connolly JL, Schnitt S J (2002) Nonmalignant lesions in breast core needle biopsies: to excise or not to excise? Am J Surg Pathol 26: 1095–1110 12218567 10.1097/00000478-200209000-00001

[bib13] Lakhani SR, Audretsch W, Cleton-Jensen A-M, Cutuli B, Ellis I, Eusebi V, Greco M, Houslton RS, Kuhl CK, Kurtz J, Palacios J, Peterse H, Rochard F, Rutgers E, on behalf of Eusoma (2006) The management of lobular carcinoma *in situ* (LCIS). Is LCIS the same as ductal carcinoma *in situ* (DCIS)? EJC 42: 2205–2211, doi: 10.1016/j.ejca.2006.03.019 10.1016/j.ejca.2006.03.019 16876991

[bib14] Lee AHS, Denley HE, Pinder SE, Ellis IO, Elston CW, Vujovic P, Macmillan RD, Evans AJ, for the Nottingham Breast Team (2003) Excision biopsy findings of patients with breast needle core biopsies reported as suspicious of malignancy (B4) or lesion of uncertain malignant potential (B3). Histopathology 42: 331–336, doi:10.1046/j.1365-2559.2003.01582.x 12653944 10.1046/j.1365-2559.2003.01582.x

[bib15] Liberman L (2000) Clinical management issues in percutaneous core breast biopsy. Radiol Clin North Am 38: 791–807 10943278 10.1016/s0033-8389(05)70201-3

[bib16] Liberman L, Tornos C, Huzjan R, Bartella L, Morris EA, Dershaw DD (2006) Is surgical excision warranted after benign, concordant diagnosis of papilloma at percutaneous breast biopsy? Am J Reontgeno 186: 1328–1334, doi: 10.2214/AJR.05.0151 10.2214/AJR.05.0151 16632727

[bib17] NHS Non-operative Diagnosis Subgroup of the National Coordination Group for Breast Screening Pathology (2001) Guidelines for Non-operative Diagnostic Procedures and Reporting in Breast Cancer Screening (NHSBSP publication no. 50). NHS Cancer Screening Programmes: Sheffield

[bib18] Parker SH, Burbank F, Jackman RJ, Aucreman CJ, Cardenosa G, Cink TM, Coscia Jr JL, Eklunk GW, Evans III WP, Garver PR (1994) Percutaneous large-core breast biopsy: a multi-institutional study. Radiology 193: 359–364 7972743 10.1148/radiology.193.2.7972743

[bib19] Perry N, Broeders M, de Wolf C, Tornberg S, Holland R, von Karsa L, Putaar E (eds) (2006) European Guidelines for Quality Assurance in Breast Cancer Screening and Diagnosis 4th edn, Europe against Cancer, European Commision, Luxembourg 10.1093/annonc/mdm481 18024988

[bib20] Shah VI, Flowers CI, Douglas-Jones AG, Dallimore NS, Rashid M (2006) Immunohistochemistry increases the accuracy of diagnosis of benign papillary lesions in breast core needle biopsy specimens. Histopathology 48: 683–691 16681684 10.1111/j.1365-2559.2006.02404.x

